# Evaluation of tibial eminence morphology using magnetic resonance imaging (MRI) in juvenile patients with complete discoid lateral meniscus

**DOI:** 10.1186/s12891-022-06002-4

**Published:** 2022-11-29

**Authors:** Wei Liu, Chunxu Fu, Kai Kang, Teng Huang, Shigang Jiang, Juyuan Gu, Shijun Gao

**Affiliations:** Department of Orthopaedic Surgery, Third Hospital of Hebei Medical University, 139 Ziqiang Road, Shijiazhuang, 050051 Hebei People’s Republic of China

**Keywords:** Discoid lateral meniscus, Tibial eminence, Magnetic resonance imaging, Juvenile

## Abstract

**Background:**

Many studies have shown that hypoplasia of knee bone morphology is related to the morphological features of the discoid lateral meniscus (DLM). However, few studies have focused on hypoplasia of tibial eminence morphology in juvenile patients with complete DLM. The purpose of this study was to determine the relationship between tibial eminence morphology characteristics and complete DLM in juvenile patients.

**Methods:**

The DLM group comprised 34 juvenile patients with complete DLM, and the control group comprised 34 juvenile individuals, each with a normal lateral meniscus based on magnetic resonance imaging (MRI) findings. All parameters, including tibial width (TW), tibial eminence width (TEW), the height of the lateral tibial spine (HLTS), the height of the medial tibial spine (HMTS), lateral slope angle of the lateral tibial eminence (LSALTE), lateral slope angle of the medial tibial eminence (LSAMTE), tibial eminence width ratio (TEWR), height of the lateral tibial spine ratio (HLTSR), and the height of the medial tibial spine ratio (HMTSR), were recorded using coronal MR images. Statistical analyses were used to determine the differences between the two groups and whether differences were significant.

**Results:**

The TEW and TEWR were significantly greater (*P* < 0.05), and LSALTE and LSAMTE were significantly smaller (*P* < 0.05) in patients in the DLM group than in participants in the control group. Receiver operating characteristic (ROC) analysis revealed that a larger TEW, above 13.4 mm, was associated with complete DLM, with a sensitivity of 77.0% and specificity of 88.2%, and a larger TEWR, above 19.7%, was associated with complete DLM, with a sensitivity of 76.5% and specificity of 91.2%.

**Conclusions:**

MR imaging can be used to diagnose tibial eminence hypoplasia in juvenile patients with complete DLM. Additionally, TEW and TEWR could help clinicians screen for complete DLM in juvenile patients.

## Background

The discoid lateral meniscus (DLM) is a congenital disorder of the knee with a thickened and discoid-shaped meniscus. The incidence of the DLM is 15% in the Asian population, which is higher than that in other populations [1]. Compared to knees with other types of meniscus, knees with complete DLM are more prone to tearing due to the larger morphology [2, 3]. The discoid meniscus tears usually show regularity, leading to different treatment from that of normal meniscus [4, 5]. The knowledge of specific bone morphology measurements suggestive of a complete DLM would be helpful to the orthopedic surgeon in case there is already a tear seen on the magnetic resonance images. According to a previous study, hypoplasia of the lateral tibial plateau and the lateral femoral condyle was impacted by the morphological features of the DLM [6].

Characteristic knee bone morphology findings of DLM, including a widened lateral joint line, cupping of the lateral tibial plateau, squaring of the lateral femoral condyle, an elevated fibular head and a condylar cut-off sign, have been reported [2, 7–12]. According to the study of Milewski et al., Asian children had 2.41 times the odds of surgery for discoid meniscus compared with Caucasian children [13]. However, as an important structure of the tibial plateau, few studies have paid attention to hypoplasia of tibial eminence morphology in juvenile patients with complete DLM. Hino et al. investigated the tibial eminence width using plain radiographs and found a wider tibial eminence width in knees with complete DLM knees [14]. However, Hino et al. did not include children under 15 years of age because it is difficult to evaluate immature bone morphology on plain radiographs. Hence, to evaluate bone morphology for patients with complete DLM, the clinical utility of plain radiographs in juvenile patients is inferior to that in adults due to skeletal immaturity. Magnetic resonance imaging (MRI) is a fundamental tool for the diagnosis of DLM and can clearly distinguish the immature bone morphology [1, 15]. Furthermore, to our knowledge, there are no studies that have evaluated tibial eminence morphology using coronal MR images in juvenile patients with complete DLM.

Therefore, the aim of this study was (1) to evaluate tibial eminence morphology in juvenile patients using parameters of coronal MR images to differentiate complete DLM compared to the characteristics of participants in the control group and (2) to determine the cut-off value of MRI parameters to differentiate complete DLM from a normal lateral meniscus. We hypothesized that there would be differences in the parameters of coronal MR images between juvenile patients with complete DLM and a normal lateral meniscus.

## Materials and methods

### Subjects

This study was approved by the institutional review board and informed consent was waived due to the retrospective study design.

Between January 2021 and May 2022, patients’ electronic charts with DLM or normal lateral meniscus in our hospital were retrospectively reviewed. According to a previous study by Samoto et al., the type of the meniscus was determined by the ratio of the minimum meniscal width to the maximum tibial width on coronal MRI view [16]. The ratio of complete DLM was > 0.32, and the ratio of normal meniscus was < 0.2. Inclusion criteria were: (1) age from 9 to 15 years and (2) MRI-confirmed complete DLM or normal lateral meniscus. Exclusion criteria were: (1) patients with suboptimal MR images (such as motion or low signal-to-noise ratio), (2) patients with knee joint bone tumors, (3) knee trauma that may affect normal bone morphology, and (4) other conditions that may affect the recognition of the knee bone morphology.

### MRI evaluations

All MRI examinations were performed using a 1.5-T (Avanto, Siemens, Erlangen, Germany) or 3.0-T MRI (Verio, Siemens, Erlangen, Germany) scanner without contrast. The measured parameters were all evaluated on coronal MR images (repetition time 3800-3900 ms; echo time 30 ms; field of view 16 cm; matrix 254 × 192-316; slice thickness 3.5 mm) with the patient supine and the knees in full extension. Accurate coronal MR images with Digital Imaging and Communications in Medicine (DICOM) were obtained using the image processing software RadiAnt DICOM Viewer (21.2 Medixant, Poznan, Poland). For both groups, the following parameters of coronal MR images were measured: tibial width (TW), tibial eminence width (TEW), the height of the lateral tibial spine (HLTS), the height of the medial tibial spine (HMTS), lateral slope angle of the lateral tibial eminence (LSALTE), and lateral slope angle of the medial tibial eminence (LSAMTE).

**Fig. 1:**
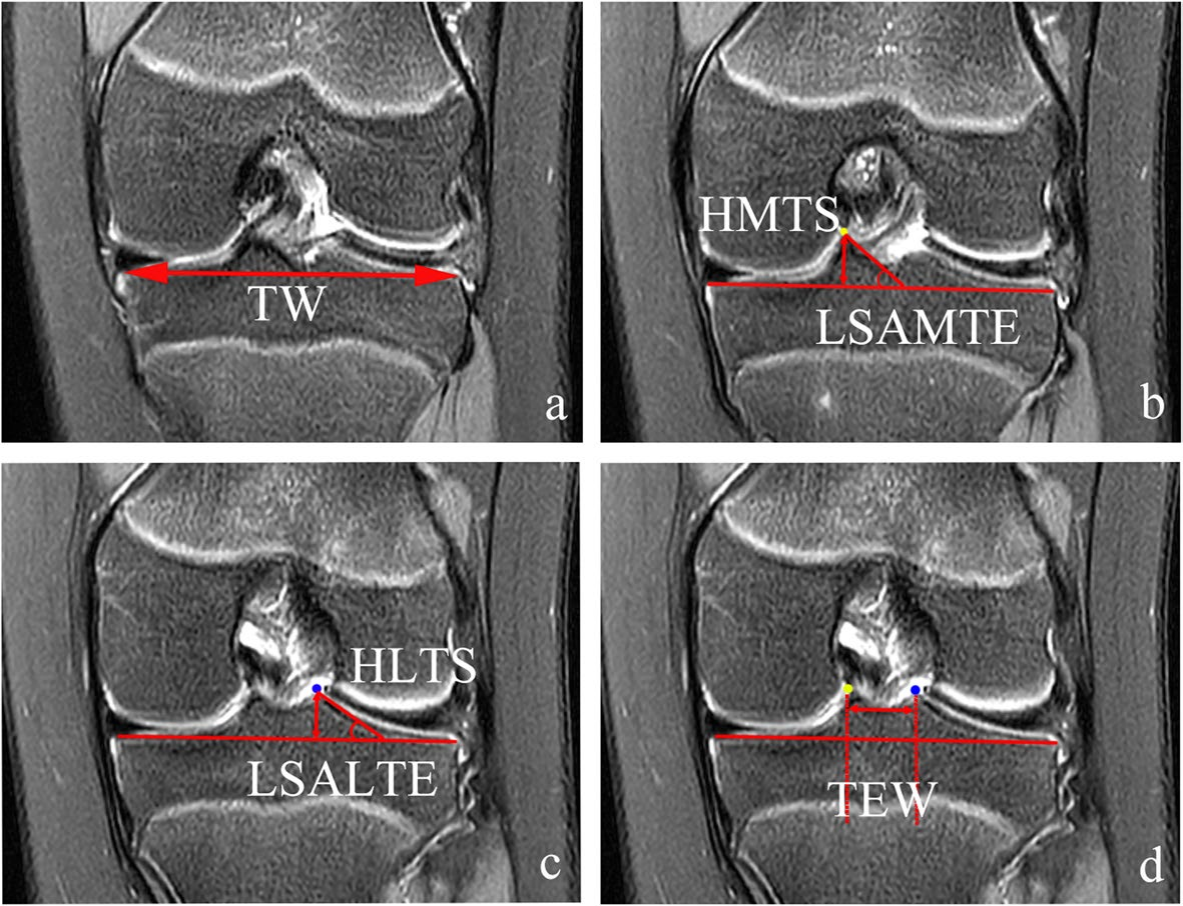
MRI parameters measured for evaluation of tibial plateau morphology. Coronal proton density-weighted MR images showing: **a** TW, maximum tibial plateau width (double-headed arrow). **b** On the slice showing the peak of the medial tibial eminence (yellow point), HMTS was the distance from the tip of the medial tibial spine to the imaginary tibial joint line (double-headed arrow), and LSAMTE was the angle between the line drawn along the lateral slope of the medial tibial spine and the imaginary tibial joint line. **c** On the slice showing the peak of the lateral tibial eminence (blue point), HLTS was the distance from the tip of the lateral tibial spine to the imaginary tibial joint line (double-headed arrow), and LSALTE was the angle between the line drawn along the lateral slope of the lateral tibial spine and the imaginary tibial joint line. **d** TEW was the distance between the peak of the lateral and the medial tibial eminence (double-headed arrow). Note that the positions of the yellow point and the blue point are different on the coronal images

The measurements of the parameters of coronal MR images are shown in Fig. 1. TW was the coronal slice showing the maximum tibial plateau width. Because the bone morphology does not appear on the same coronal slice in three dimensions, the peaks of the lateral and the medial tibial eminence were marked in multiplanar images. TEW was the distance between the peak of the lateral and the medial tibial eminence. On the slice showing the peak of the lateral tibial eminence, HLTS was the distance from the tip of the lateral tibial spine to the imaginary tibial joint line, and LSALTE was the angle between the line drawn along the lateral slope of the lateral tibial spine and the imaginary tibial joint line. On the slice showing the peak of the medial tibial eminence, HMTS was the distance from the tip of the medial tibial spine to the imaginary tibial joint line, and LSAMTE was the angle between the line drawn along the lateral slope of the medial tibial spine and the imaginary tibial joint line. In addition, the tibial eminence width ratio (TEWR) was defined as that the TEW was divided by the TW and multiplied by 100%. The height of the lateral tibial spine ratio (HLTSR) was defined as that the HLTS was divided by the TW and multiplied by 100%. The height of the medial tibial spine ratio (HMTSR) was defined as that the HMTS was divided by the TW and multiplied by 100%. Two experienced orthopaedic surgeons (6 and 8 years of clinical experience, respectively) who were blinded to any clinical information performed the measurements on RadiAnt DICOM Viewer. Each parameter was measured twice with at least a two-week interval.

### Statistical analysis

Statistical analysis was performed with IBM SPSS Statistics Version 26 (SPSS, Chicago, IL). Statistical significance was set at 𝑃 < 0.05. The obtained test results were described as the mean ± standard deviation, range or frequency (%). The Shapiro-Wilk normality test and the Levene statistic were used to analyse normality of distribution and the homogeneity of variance of the data. According to statistical distribution, the Mann-Whitney U test and the independent-samples T test were used for comparison of all continuous variables, including age, weight, height, body mass index (BMI), TW, TEW, HLTS, HMTS, LSALTE, LSAMTE, TEWR, HLTSR, HMTSR between participants in the complete DLM group and the control group. The chi-square test was used for comparison of categorical variables, including gender and laterality, between participants in the two groups. The threshold points of each statistically significant coronal MRI parameter were determined by the receiver operating characteristic (ROC) curve and the area under the curve (AUC) to distinguish between participants in the two groups. The cut-off value was derived from the point with the maximal Youden index, which corresponds to the highest sum of sensitivity and specificity. The power of each statistically significant coronal MRI parameter was analysed in Pass software (15.0.5, NCSS, LLC, Kaysville, UT). The intraobserver and interobserver reliabilities were calculated by intraclass correlation coefficients (ICCs).

## Results

**Fig. 2:**
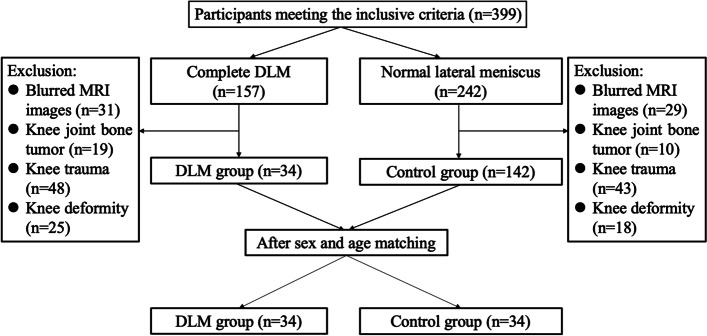
Flowchart of participants enrolment

After reviewing patients’ coronal MR images, 34 complete DLM cases were included in present study. In addition, 34 juvenile age- and gender- matched patients from the patient pool of our hospital who were confirmed to have a normal lateral meniscus were enrolled in this study as a control group. The flowchart of the patient enrolment is shown in Fig. 2. There was no significant difference in demographics between participants in the two groups. The demographic characteristics of the subjects are presented in Table 1.

**Table 1:** Demographic characteristics of the subjects

	DLM Group (*n* = 34)	Control Group (*n* = 34)	*P* value
Age, y	12.8 ± 1.6	12.9 ± 1.7	0.836^b^
Height, cm	151.9 ± 13.6	148.0 ± 12.4	0.239^a^
Weight, kg	50.5 ± 12.6	47.3 ± 12.0	0.239^a^
BMI, kg/m^2^ Laterality, left/right	21.5 ± 1.9 23/11	21.1 ± 2.2 16/18	0.932^a^ 0.086^c^
Gender, male/female	20/14	20/14	

Data was given as n or mean ± standard deviation. Significance was calculated using a two-tailed

^a^2 independent-samples t-tests

^b^Mann-Whitney U test

^c^chi-square test

All parameters of coronal MR images measured in the two groups are summarized in Table 2. Comparing the analysis of participants in the complete DLM group and the control group, the Mann-Whitney U test and the independent-samples T test showed statistically significant differences (*P* < 0.05) in TEW, LSALTE, LSAMTE, and TEWR. The mean TEW and TEWR were 14.9 ± 1.9 mm and 22.0 ± 2.8% for participants in the complete DLM group, respectively, which were significantly wider than those (11.8 ± 1.7 mm and 17.4 ± 2.0%, respectively) of participants in the control group (*P* < 0.05). The mean LSALTE and LSAMTE were 26.2 ± 5.3 ° and 31.5 ± 4.4 ° for participants in the complete DLM group, respectively, which were significantly smaller than those (34.0 ± 6.3 ° and 37.6 ± 6.3 °, respectively) for participants in the control group (*P* < 0.05). For an *α* < 0.05, the powers of TEW, LSALTE, LSAMTE, and TEWR were 100, 99, 99 and 100%, respectively. We also found smaller means for TW, HLTS, HLTSR (67.8 ± 5.6 mm, 7.6 ± 1.4 mm, 11.2 ± 1.9%, respectively) and larger mean of HMTS, HMTSR (9.1 ± 1.5 mm, 13.4 ± 1.9%, respectively) in participants in the complete DLM group compared those (68.1 ± 6.9 mm, 8.1 ± 1.1 mm, 12.0 ± 2.0%, 8.8 ± 1.2 mm, 13.0 ± 1.4%, respectively) for participants in the control group. However, TW, HLTS, HLTSR, HMTS, and HMTSR did not show statistically significant differences between participants in the two groups (*P* > 0.05).

**Table 2:** Comparison of each measurement between groups

variable	DLM Group (*n* = 34)	Control Group (*n* = 34)	*P* value
TW, mm	67.8 ± 5.6	68.1 ± 6.9	0.842^a^
TEW, mm	14.9 ± 1.9	11.8 ± 1.7	< 0.001^a*^
HLTS, mm	7.6 ± 1.4	8.1 ± 1.1	0.108^a^
HMTS, mm	9.1 ± 1.5	8.8 ± 1.2	0.461^a^
LSALTE, mm	26.2 ± 5.3	34.0 ± 6.3	< 0.001^b*^
LSAMTE, mm	31.5 ± 4.4	37.6 ± 6.3	< 0.001^a*^
TEWR, %	22.0 ± 2.8	17.4 ± 2.0	< 0.001^a*^
HLTSR, %	11.2 ± 1.9	12.0 ± 2.0	0.089^a^
HMTSR, %	13.4 ± 1.9	13.0 ± 1.4	0.348^a^

All data was given as mean ± standard deviation. Significance was calculated using a two-tailed

^*^Significant difference

^a^2 independent-samples t-tests

^b^Mann-Whitney U test

**Fig. 3:**
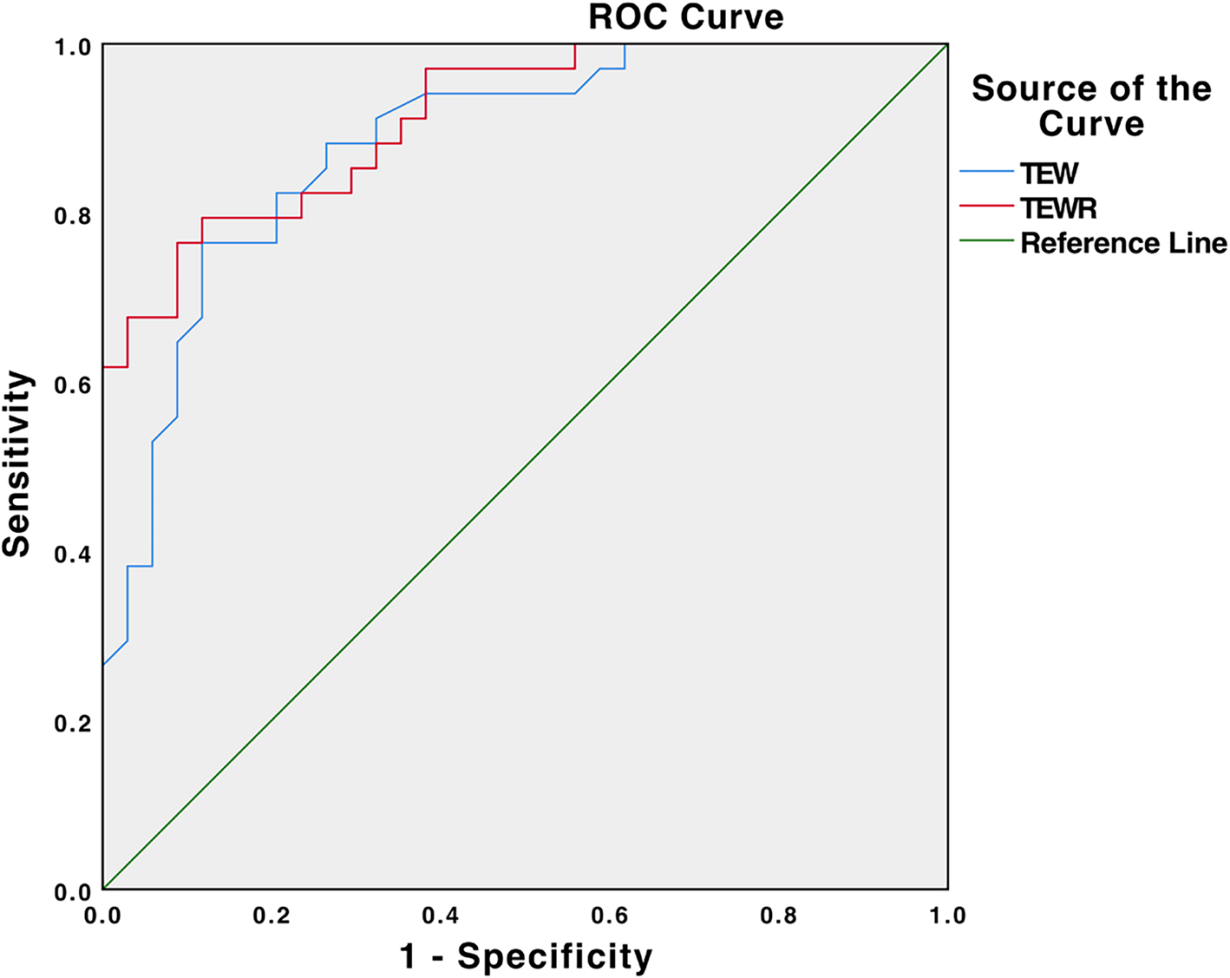
ROC curve analysis was performed to determine the thresholds of TEW and TEWR that were associated with complete DLM

The ROC curve analysis of TEW resulted in an AUC of 0.883 with a cut-off value setting at 13.4 mm (Youden index 0.652), yielding a sensitivity of 77.0% and specificity of 88.2% for predicting complete DLM. The ROC curve analysis of TEWR produced an AUC of 0.913 with a cut-off value setting at 19.7% (Youden index 0.677), yielding a sensitivity of 76.5% and specificity of 91.2% for predicting complete DLM (Table 3, Fig. 3). The ROC curve analysis of LSALTE and LSAMTE produced an AUCs of 0.165 and 0.213, respectively, which indicated a noninformative test for predicting complete DLM. In addition, increased TEWR (> 19.7%) was determined to be a risk factor for complete DLM (OR = 2.073, 95%CI = 1.217 to 3.528).

**Table 3:** Cut-off values and coordinates of the ROC curves

variable	AUC (95% CI)	Cut-off values	Sensibility, %	Specificity, %	*P* value
TEW	0.883 (0.804-0.962)	13.4 mm	77.0	88.2	< 0.001^a^
TEWR	0.913 (0.849-0.977)	19.7%	76.5	91.2	< 0.001^a^

The cut-off was determined at the maximal Youden index

^a^Significant difference

All measurements in this study were highly reliable, with ICCs values ranging from 0.849 to 0.989, regardless of the two observers and the interval of observation.

## Discussion

The most important finding of the present study is that higher TEW and TEWR values were reliable parameters of coronal MR images to predict the diagnosis of complete DLM in juvenile patients which was in agreement with our hypotheses. The cut-off value of 13.4 mm for TEW could diagnose complete DLM with a sensitivity of 77.0% and specificity of 88.2%. The cut-off value of 19.7% for TEWR could diagnose complete DLM with a sensitivity of 76.5% and specificity of 91.2%. These knee bone morphology characteristics are useful for orthopedic surgeons to infer the type of meniscus in juvenile patients in case that a DLM may not be clearly seen by using MRI, such as there is already a tear seen on the MR image. Because, firstly, the treatment of discoid meniscus tears is usually different from that of normal meniscus, and discoid meniscus tears usually show regularity. Niu et al. reported that saucerization is currently regarded as favored treatment for patients with discoid meniscus. After saucerization, peripheral rim instability may be managed by various standard meniscal repair techniques [4]. Klingele et al. reported that peripheral rim tears and instability patterns in discoid meniscus have been noted most commonly in the anterior horn (47.2%), followed by the posterior horn (38.9%) and middle third (11.1%) [5]. So, preoperative diagnosis of complete DLM is important for orthopedic surgeons to perfect the preoperative plan and shorten the operation time. Secondly, the discoid meniscus is more prone to tearing than normal meniscus and the incidence of bilateral discoid meniscus has been reported between 15 and 25% [2, 3, 5, 17]. Patel et al. reported that young patients with unilateral symptomatic discoid meniscus have been found to be 4.5 times more likely to eventually require bilateral surgical intervention [18]. So, the diagnosis of unilateral discoid meniscus may prompt the orthopedic surgeon to pay more attention to the contralateral meniscus to reduce the risk of meniscal tears. Thirdly, Kocher et al. reported that compared to physical examination, MRI has a lower sensitivity for diagnosing DLM [19]. This study added more reliable morphologic signs which could help the orthopedic surgeon to improve the accuracy of diagnosis of complete DLM by using MRI. Additionally, TEWR was a significant risk factor for complete DLM, and smaller LSALTE and LSAMTE were significantly related to complete DLM.

MRI is a more expensive and complex method than radiographic methods. In addition, MRI has contraindications for patients with claustrophobia [20]. Despite these limitations, MRI is a useful method to distinguish the immature bone morphology in juvenile patients [15]. Previous studies have reported that many radiographic parameters, including a widened lateral joint line, cupping of the lateral tibial plateau, squaring of the lateral femoral condyle, an elevated fibular head and a condylar cut-off sign, are useful and convenient for DLM screening [2, 7–12]. Arthroscopic visualization and MRI parameters are also utilized in DLM screening [6, 21–23]. All these parameters originate from the hypoplasia of the lateral tibial plateau and the lateral femoral condyle related to DLM. However, few studies have focused on the hypoplasia of the tibial eminence morphology in complete DLM patients. Hino et al. investigated the tibial eminence width in patients with complete DLM and failed to include children younger than 15 years because of the difficulties in evaluating the tibial eminence morphology of juvenile patients by using plain radiographs [14]. The authors found higher TEW and TEWR values in patients with complete DLM (15.8 ± 3.1 mm, 21.8 ± 2.7%, respectively) compared to those of people with a normal lateral meniscus (12.6 ± 1.2 mm, 16.7 ± 1.7%, respectively), which agreed with our results (14.9 ± 1.9 mm, 22.0 ± 2.8% to 11.8 ± 1.7 mm, 17.4 ± 2.0%, respectively). A possible explanation for the higher TEW and TEWR values is that the thickened and discoid-shaped meniscus impacts the tibial eminence morphology, leading to hypoplasia of the lateral tibial eminence. The cut-off value of TEW was 13.9 mm in their study, which was slightly wider than 13.4 mm in this study. The cut-off value of TEWR was 18.8% in their study, which was slightly smaller than the value of 19.7% in this study. A possible explanation for the difference is that the tibial eminence width of juvenile patients is not fully developed but is relatively larger than that of older patients. Therefore, TEWR could be a risk factor for complete DLM in juvenile patients in our study. The authors of the previous study also reported higher sensitivity and lower specificity of TEW and TEWR (100 and 83%, 100 and 90%, respectively) compared to the values in our study (77 and 88.2%, 76.5 and 91.2%, respectively). Although different subjects and materials may impact the sensitivity and specificity of the results, both studies acquired excellent predictors of complete DLM.

We also found significant differences in LSALTE and LSAMTE values between the two groups in this study. However, according to the ROC curve analysis, the AUCs of LSALTE and LSAMTE (0.165 and 0.213, respectively) were low and could not be reliable predictors of complete DLM in juvenile patients. Park et al. performed a retrospective study to investigate the diagnostic accuracy of radiographic signs for complete DLM in adults [2]. They found a significantly smaller LSALTE in participants in the complete DLM group than in participants in the control group (32.1 ± 6.7 ° to 35.2 ± 5.2 °, *P* = 0.000). In another study, Park et al. reported a significantly smaller LSALTE in participants in the complete DLM group than in participants in the control group (31.57 ° [29.78 °-32.39 °] to 33.38 ° [32.21 °-34.14 °], *P* = 0.0339) in children aged 10 to 16 years [7]. Hino et al. investigated the LSAMTE of knees with DLM on plain radiographs and found a significantly smaller LSAMTE in participants in the complete DLM group than in participants in the normal lateral meniscus group (23.6 ± 4.2 ° to 28.6 ± 3.0 °, *P* < 0.05) [14]. The results of this study, which also found significantly smaller LSALTE and LSAMTE in participants in the complete DLM group than in participants in the control group (26.2 ± 5.3 ° to 34.0 ± 6.3 ° and 31.5 ± 4.4 ° to 37.6 ± 6.3 °, respectively), are similar to those of previous studies. The results could illustrate the hypoplasia of the tibial eminence in juvenile patients with complete DLM. A possible explanation for the lower values of the AUCs of LSALTE and LSAMTE is that the thickened and discoid-shaped DLM results in great variation in LSALTE and LSAMTE, leading to not reliable AUC in the ROC curve analysis.

We did not find significant differences in TW, HLTS, HLTSR, HMTS, or HMTSR between participants in the two groups in this study. Hino et al. did not report the differences in TW between participants in the complete DLM group and the normal lateral meniscus group [14]. In their study, Jiang et al. investigated the HLTS and HMTS of DLM patients, who had a median age of 41 years [9]. The authors found a significantly smaller HLTS in participants in the DLM group than in participants in the control group (6.7 ± 2.0 mm to 7.9 ± 1.8 mm, *P* < 0.05) and did not find significant differences in HMTS between participants in the two groups. Song et al .[11] reported smaller HLTS in participants in the DLM group compared to that of participants in the control group (7.2 ± 1.6 mm to 7.8 ± 1.6 mm), but the authors did not find significant differences in the HLTS between the two groups, which was similar to our results (7.6 ± 1.4 mm, to 8.1 ± 1.1 mm).

Our study had several limitations. First, this study was retrospective in nature in a single centre, which may be due to the results. Although all subjects in this study were Chinese, we still consider our results valuable because of the high incidence of the DLM in Asians. Second, we did not include patients with incomplete DLM in our study due to the lower morbidity in clinical problems in these individuals compared to that of patients in the complete DLM group [24, 25]. Further multicenter research to confirm the results are needed in the future.

## Conclusions

This study demonstrates that hypoplasia of tibial eminence is found on coronal MR images based on the parameters of TEW, LSALTE, LSAMTE, and TEWR in juvenile patients with complete DLM. Additionally, the results of TEW and TEWR would be useful for screening complete DLM in juvenile patients.

## Data Availability

All data and materials are available from the corresponding author upon request.

## Abbreviations

DLM: Discoid lateral meniscus
MRI: Magnetic resonance imaging
TW: Tibial width
TEW: Tibial eminence width
HLTS: Height of the lateral tibial spine
HMTS: Height of the medial tibial spine
LSALTE: Lateral slope angle of the lateral tibial eminence
LSAMTE: Lateral slope angle of the medial tibial eminence
TEWR: Tibial eminence width ratio
HLTSR: Height of the lateral tibial spine ratio
HMTSR: Height of the medial tibial spine ratio
ROC: Receiver operating characteristic
DICOM: Digital Imaging and Communications in Medicine
BMI: Body mass index
AUC: Area under the curve
ICCs: Intraclass correlation coefficients
